# Identification of anoikis-related subtypes and immune landscape in kidney renal clear cell carcinoma

**DOI:** 10.1038/s41598-023-45069-4

**Published:** 2023-10-23

**Authors:** Wencong Ding, Min Zhang, Ping Zhang, Xianghong Zhang, Junwei Sun, Biying Lin

**Affiliations:** The Department of Nephrology and Hemopurification Center, Affiliated Guangdong Hospital of Integrated Traditional Chinese and Western Medicine of Guangzhou University of Chinese Medicine, Foshan, 528000 Guangdong China

**Keywords:** Cancer, Computational biology and bioinformatics

## Abstract

Anoikis is a specific form of programmed cell death induced by the loss of cell contact with the extracellular matrix and other cells, and plays an important role in organism development, tissue homeostasis, disease development and tumor metastasis. We comprehensively investigated the expression patterns of anoikis-related genes (ARGs) in kidney renal clear cell carcinoma (KIRC) from public databases. Anoikis-related prognostic signatures were established based on four ARGs expression, in which KIRC patients were assigned different risk scores and divided into two different risk groups. In addition, four ARGs expression was validated by qRT-PCR. A better prognosis was observed in the low-risk group, but with lower immune activity (including immune cells and immune-related functions) in the tumor microenvironment. Combined with the relevant clinical characteristics, a nomogram for clinical application was established. Receiver operating characteristics (ROC) and calibration curves were constructed to demonstrate the predictive power of this risk signature. In addition, higher risk scores were significantly and positively correlated with higher gene expression of tumor mutation load (TMB), immune checkpoints (ICPs) and mismatch repair (MMR)-related proteins in general. The results also suggested that the high-risk group was more sensitive to immunotherapy and certain chemotherapeutic agents. Anoikis-related prognostic signatures may provide a better understanding of the roles of ARGs and offer new perspectives for clinical prognosis and individualized treatment.

## Introduction

Renal cell carcinoma (RCC) is one of the most common types of cancer in humans and is classified into three main subtypes: renal clear cell carcinoma (KIRC), renal papillary cell carcinoma (KIRP), and suspicious cell malignancy^1^. Of these, renal clear cell carcinoma (KIRC) is the most prevalent subtype^2^. Moreover, KIRC patients usually do not exhibit obvious symptoms in the early stages, and approximately 30% of KIRC cases show metastases upon detection. Currently, surgical resection remains the most effective treatment for KIRC patients^3^. However, the prognosis of KIRC is still unsatisfactory due to its high recurrence rate^4^. While PD-1/PD-L1 blockers have been approved for the treatment of KIRC, some patients still respond poorly and show resistance to progression^5^. In addition, RCC is essentially a metabolic disease characterized by reprogramming of energy metabolism^6–9^. The metabolic flux of glycolysis in RCC patients is partitioned^10–12^, especially the impaired mitochondrial bioenergy, oxidative phosphorylation, and lipid metabolism^13–15^. The use of comprehensive next-generation sequencing methods to better understand ccRCC can help define and predict its behavior in terms of invasiveness, prognosis, and treatment response, and will become an innovative strategy for selecting the best treatment for specific patients. Thus, it is critical to unveil the underlying mechanisms of KIRC and establish an accurate prognostic model for diagnosis and treatment strategies for kidney cancer.

Anoikis is a specific form of programmed cell death induced by the loss of cell contact with the extracellular matrix and other cells^16,17^. Anoikis occurs after the disruption of cell-extracellular matrix (ECM) interactions and is required for tumor cell survival after separation from the extracellular matrix. The emergence of anoikis resistance in tumors with loss of nesting can help isolated tumor cells avoid death signaling pathways and allow cell survival under unfavorable conditions^18^. Anoikis resistance has been reported in various cancers, including lung cancer, where the PLAG1–GDH1 axis promotes anoikis resistance and tumor metastasis through CamKK2-AMPK signaling^19^. Several synthetics have been shown to exhibit pro-apoptotic potential in lung cancer cells and in vivo models to aid in the clinical management of patients^20^. Although the development of anoikis resistance has been associated with metastasis in various cancers, studies on anoikis in KIRC are rare.

Thus, this study focused on the predictive performance of anoikis-related genes (ARGs) in prognosis of KIRC and developed an anoikis-associated risk score model by univariate cox analysis and least absolute shrinkage and selection operator (lasso) analysis. This study further explored and compared differences in gene mutations, functional enrichment, and immune microenvironment between the two risk groups. The prognostic role of anoikis in the clinic has been used to provide a basis for individualized treatment of KIRC patients.

## Materials and methods

### Data acquisition

RNA sequence transcriptome data, mutations and clinical data of KIRC patients were downloaded from The Cancer Genome Atlas (TCGA, https://portal.gdc.cancer). RNA-Seq data included 542 tumor samples and 72 normal samples, and after excluding samples with incomplete prognostic information, a total of 533 KIRC patients were screened for further analysis. The exclusion criteria included removing all samples without clinical follow-up information, removing all samples with the unknown survival time, and removing all samples without a survival status. As an external validation dataset, 39 samples from GSE29609 were obtained from the Gene Expression Omnibus (https://www.ncbi.nlm.nih.gov, GEO) database. We obtained 434 Anoikis-associated genes (ARGs) from previously published articles^21^. The “limma” package was used to screen the TCGA-KIRC dataset for differentially expressed genes (DEGs) and cut-off values were |log fold change (logFC)| > 1 and p < 0.05^22^. The anoikis-related genes associated with the survival of KIRC patients were identified using univariate Cox regression analysis, with a threshold of* p* < 0.05 and named as prognosis-related genes^23^.

### Functional and gene set enrichment analysis

Gene Ontology (GO) functional and Kyoto Gene and Genome Encyclopedia (KEGG) pathway enrichment analyses were performed using the “clusterProfiler” package to reveal the signaling pathways and functions of different risk groups^24^. In addition, gene set enrichment analysis (GSEA) was performed to identify biological processes between two risk groups based on the file ““c2.cp.kegg.v6.2.symbols.gmts” from the MSigDB database. Significance was determined based on a threshold of* p* < 0.05^25^.

### Immunophenoscore (IPS) and chemotherapy analysis

The immunophenotype score (IPS) is a validated predictor of response to immunotherapy against CTLA-4 and PD-1^26^. The half-maximal inhibitory concentration (IC 50) of representative drugs was assessed by a database called Genomics of Drug Sensitivity in Cancer (GDSC)^27^. We calculated the half-inhibitory concentration (IC50) values of commonly used chemotherapeutic drugs in OC using the “pRRophetic” package to examine the change in efficacy of chemotherapeutic drugs between the two groups of patients^28^. The correlation between ARGs expression and drug sensitivity was estimated in NCI-60 database by Pearson correlation analysis^29^.

### Construction of risk score signature

KIRC patients were randomly divided into three groups, including the training, testing, and entire groups in a 1:1 ratio, and the results of the chi-square test showed no significant differences between the subgroups (Supplementary Table 1). Firstly, univariate Cox regression analysis was performed on the training set to identify differently expressed ARGs associated with prognosis. The LASSO Cox regression model was used to narrow the most robust anoikis-related genes for prognosis and ten-fold cross validation was applied to overcome the over-fitting by the package “glmnet”. Four candidate genes (ITGA6, AR, PLK1 and IRF6) were subsequently obtained using multivariate Cox regression analysis. Risk scores were calculated for each sample by using the expression values of key genes and weighting their corresponding coefficients. The risk score was calculated as follows. Risk score = ∑coef ∗ Exp(genes). coef: coefficient of the gene; Exp(genes): expression of the gene. KIRC patients with different risk scores were divided into two risk groups according to the formula for calculating the median risk score. Subsequently, Kaplan–Meier survival analysis was used to reveal the prognostic differences between the two risk groups. Univariate and multivariate Cox regression analyses were performed to determine whether anoikis-related signature of lost nests could be an independent prognostic factor in KIRC patients. Based on age, grade, stage and risk score, a nomogram was created using the 'rms' R package to predict OS in clinical patients at 1, 3 and 5 years. The receiver operating characteristic (ROC) curve was evaluated the predictive ability of the signature and assess the proportional hazard. Calibration curves were generated to assess the agreement between predicted and actual survival.

### Identification of anoikis-related prognostic signature

TME is mainly composed of tumor cells, immune cells, stromal cells and extracellular matrix and the “ESTIMATE” algorithm was used to explore the cell scores of different risk groups^30,31^. The “ssGSEA” R script was used to quantify the relative proportion of infiltrating immune cells. Differences between tumor-infiltrating immune cells (TIIC) were assessed using multiple databases (TIMER, CIBERSORT, CIBERSORT-ABS, QUANTISSEQ, MCPCOUNTER, XCELL and EPIC) and the 21 TIIC components between the two risk groups were evaluated by the CIBERSORT algorithm^32^. Pearson correlation analysis showed a correlation between risk scores, ARGs expression and TIIC.

### Somatic mutation analysis

Somatic variant data were stored in mutation annotation format (MAF), and we used “maftools” to analyze mutation data from KIRC samples^33^. For each KIRC patient, we determined the tumor mutation burden (TMB) score and investigated the association between risk score and TMB. It was determined how to calculate the TMB score: (total mutations/total coverage base) × 10^6^^34^. The prognostic value of TMB in OC was investigated using Kaplan–Meier analysis.

### Cell culture and reverse transcription and PCR analysis

We acquired two types of human ccRCC cell lines (786-O and ACHN) and a human renal proximal convoluted tubule cell line (HK2) from the American Type Cultural Collection (ATCC). The 786-O cells were cultured in RPMI 1640 media (Gibco, USA), while HK2 and ACHN were maintained in DMEM high glucose media (Gibco, USA). All cell types were kept in an incubator at 37 °C with 5% CO_2_. The total RNA was extracted from the cells by using the Trizol reagent (Vazyme, China). The integrity and concentration of the extracted RNA were measured with the NanoDrop spectrophotometer (Thermo Fisher Scientific, USA). Subsequently, the total RNA was reversely transcribed to cDNA by using the RevertAid RT Kit (K1691, Thermo Fisher Scientific, USA). The cDNA was amplified with specific primers to detect target mRNA expression using qPCR Mix (RR430S, Takara, Japan) by an ABI 7500 system (Applied Biosystems, CA, USA). GAPDH was used as an internal reference. The sequences are listed in Supplementary Table 1.

### Statistical analysis

Statistical analyses were performed using R software version 4.1.3. Differences between two risk groups were calculated by Student’s *t*-test or Chi-squared test. Kaplan–Meier analysis was used to calculate differences in overall survival (OS). Relationship analysis was calculated by Pearson correlation test. The Shapiro Wilk test was used to detect whether two variables belong to a normal distribution. Benjamini–Hochberg method was used to correct for false discovery rate (FDR). The p-value and FDR < 0.05 were considered statistically significant.

## Results

### Differential expression of anoikis-related genes

**Figure 1:**
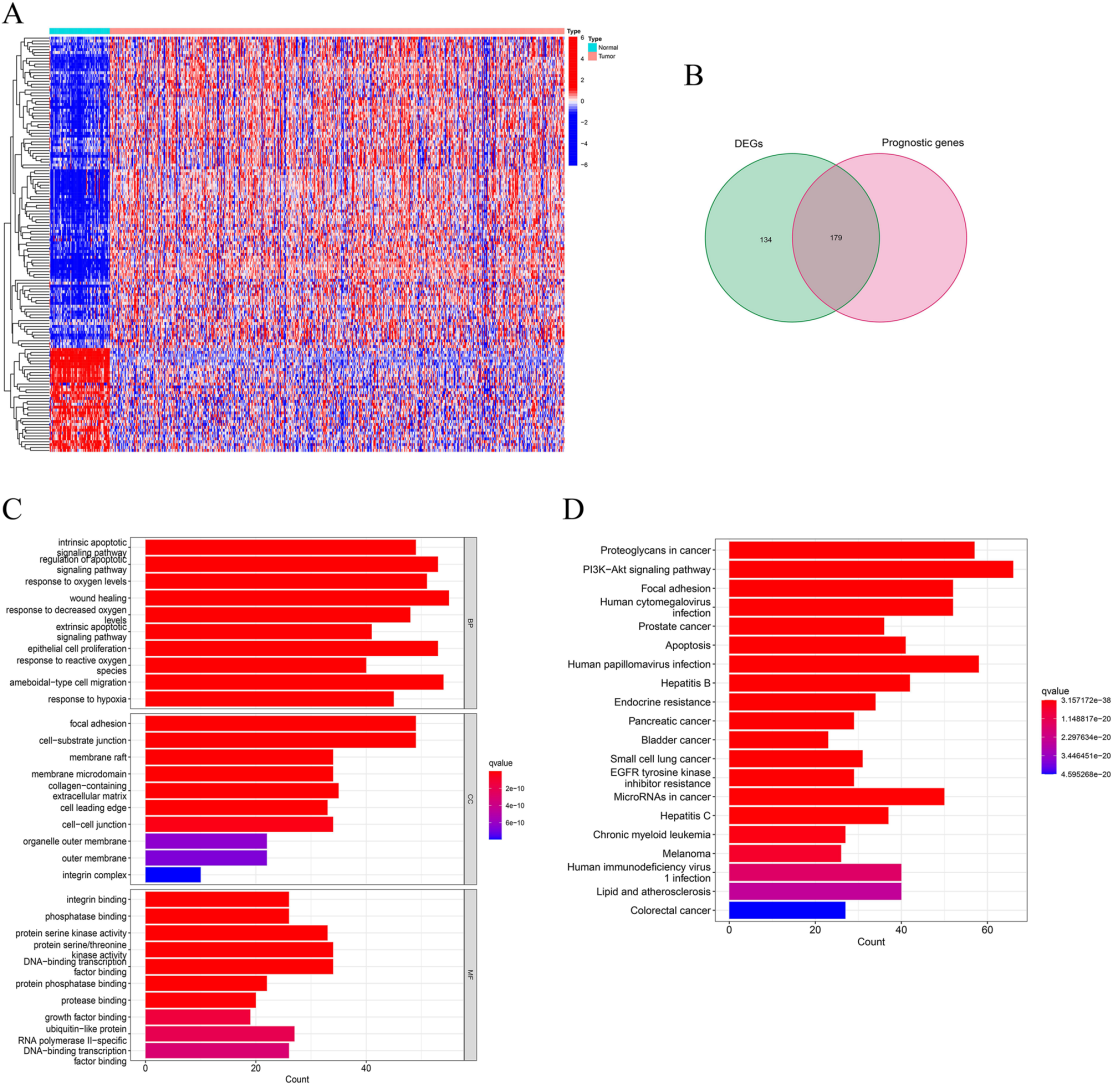
Differential expression analysis and functional analysis. (**A**) The heat map shows the difference in the expression of anoikis-related genes (ARGs) between tumor tissue and normal tissue. The blue square represents the lower expression, while the red square represents the higher expression. (**B**) The Venn diagram shows the distribution of differentially expressed genes (DEGs) and prognosis-related ARGs in patients with KIRC. (**C**,**D**) GO terms and KEGG pathway enriched analysis of differentially expressed ARGs.

Considering the potential relationship between ARGs and KIRC, first, we performed differential expression analysis in the TCGA-KIRC cohort and the 313 DEGs were exhibited in heat maps (Fig. 1A). A total of 179 intersecting genes were obtained between prognosis-related genes and DEGs (Fig. 1B). After that, we explored the biological functions of these 179 differentially expressed ARGs. GO enrichment analysis indicated that these DEGs were involved in intrinsic apoptotic signaling pathway, regulation of apoptotic signaling pathway, focal adhesion, cell-substrate junction, integrin binding, phosphatase binding (Fig. 1C). In addition, KEGG pathway analysis demonstrated that the DEGs were mainly involved in proteoglycans in cancer, PI3K-Akt signaling pathway (Fig. 1D).

### Construction of anoikis-related signatures

**Figure 2:**
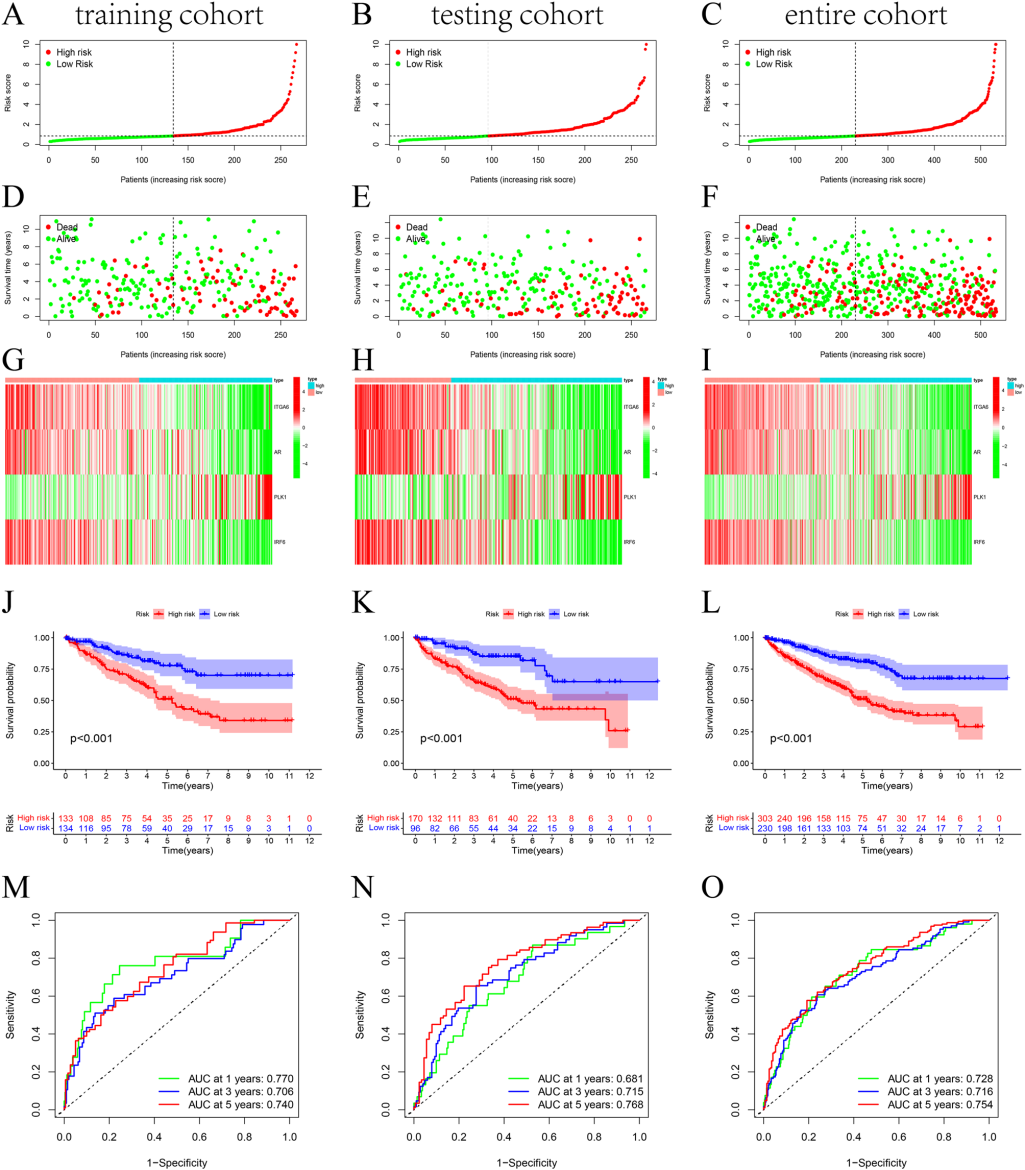
Construction and validation of anoikis-related signatures in training, testing, and the entire groups. The risk curve shows the distribution of risk scores between the high-risk and low-risk groups (**A**–**C**). Scatter plots show survival status and survival time (**D**–**F**), while heatmaps show the expression of four ARGs in high-risk and low-risk groups (**G**–**I**). Kapan Meier survival curves for overall survival (OS) of patients in the high-risk and low-risk groups (**J**–**L**). ROC analysis for predicting prognosis role of risk scores (**M**–**O**).

To investigate the prognostic role of 179 DEGs, KIRC patients were randomly divided into a training set, testing set, and entire cohort and there was no difference in clinical information among the three sets (Supplementary Table 1). In the training set, univariate cox regression analysis revealed 143 ARGs identified as associated with overall survival (OS). To construct novel anoikis-related prognostic signature, LASSO and multivariate Cox regression analyses were performed to identify the key genes (Supplementary Fig. 1). Finally, ITGA6, AR, PLK1, and IRF6 were included in this signature, and anoikis-related risk scores for KIRC patients were calculated as follows: Risk score = (− 0.216 × ITGA6 expression) + (− 0.262 × AR expression) + (0.271 × PLK1 expression) + (− 0.17 × IRF6 expression). In the training set, we ranked the patients in training set according risk score and divided the patients in training set and validation set into high risk (HR) group and low risk (LR) group according to the median value of the score. In addition, scatter plots and survival distribution plots show the survival time and survival status of KIRC patients, while heat maps show the expression levels of four genes in training set (Fig. 2A,D,G), testing set (Fig. 2B,E,H) and entire set (Fig. 2C,F,I). Kaplan Meier analysis showed that three sets showed significantly worse survival outcomes for HR patients compared to the LR group (Fig. 2J–L). In addition, the area under the curve (AUC) of 1-, 3- and 5-year OS were all greater than 0.7, indicating a high predictive sensitivity of anoikis-related prognostic signature (Fig. 2M–O). To validate the predictive ability of this prognostic signature, we download the corresponding expression profile and follow-up file from GEO database, and GSE29609 dataset was selected as the verification cohorts. As shown in Supplementary Fig. 2A,B, patients were divided into two risk groups and their survival state in different risk group was revealed. The heat map displayed the expression of four candidate ARGs in different risk groups (Supplementary Fig. 2C). In addition, the OS of patients in high-risk group were worse and the ROC curve also proved that this signature has good predictive ability for prognosis (Supplementary Fig. 2E,F). In addition, human ccRcc cell lines were used to detect the expression level of four ARGs by qRT-PCR. Compared to the human renal proximal convoluted tubule cell HK2 cells, AR expression was up-regulated in 786-O and ACHN, PLK1 expression was higher in ACHN, while there was no significant different in expression of IRF6 and ITGA6 (Supplementary Fig. 3).

### Validation of the predictive power of anoikis-related prognostic signatures in KIRC

**Figure 3:**
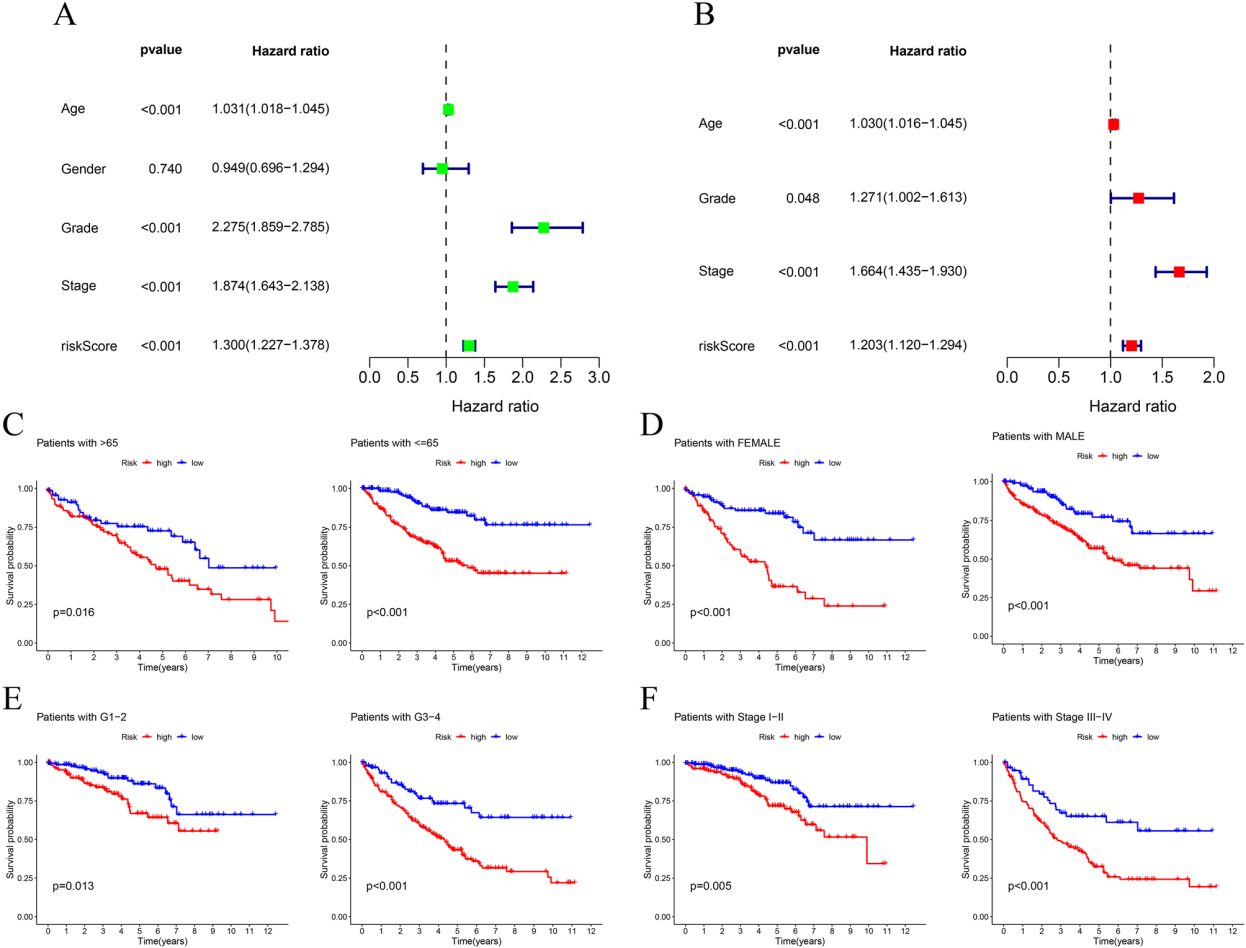
Subgroup analysis of the prognostic value of risk score. Independent factors analysis through univariate (**A**) and multivariate (**B**) Cox regression analysis. The prognostic value of risk scores for KIRC patients with different ages (**C**), genders (**D**), grades (**E**), and stages.

To verify the predictive ability of this signature, we conducted univariate cox analysis and univariate cox analysis in entire set. The results showed that risk scores, as well as age and stage, were predictive factors for better survival of patients with KIRC (Fig. 3A,B). In addition, we also conducted survival analysis in different subgroups to evaluate the predictive value of this signature. Kaplan Meier analysis showed that in KIRC patients with male or female, young or elderly patients, Grade1 and 2 or Grade3 and 4, stage I and II, or stage III and IV, the LR group had better OS (Fig. 3C–F). In summary, these results indicated that the anoikis-related risk model was a promising prognostic classification tool for patients with KIRC.

**Figure 4:**
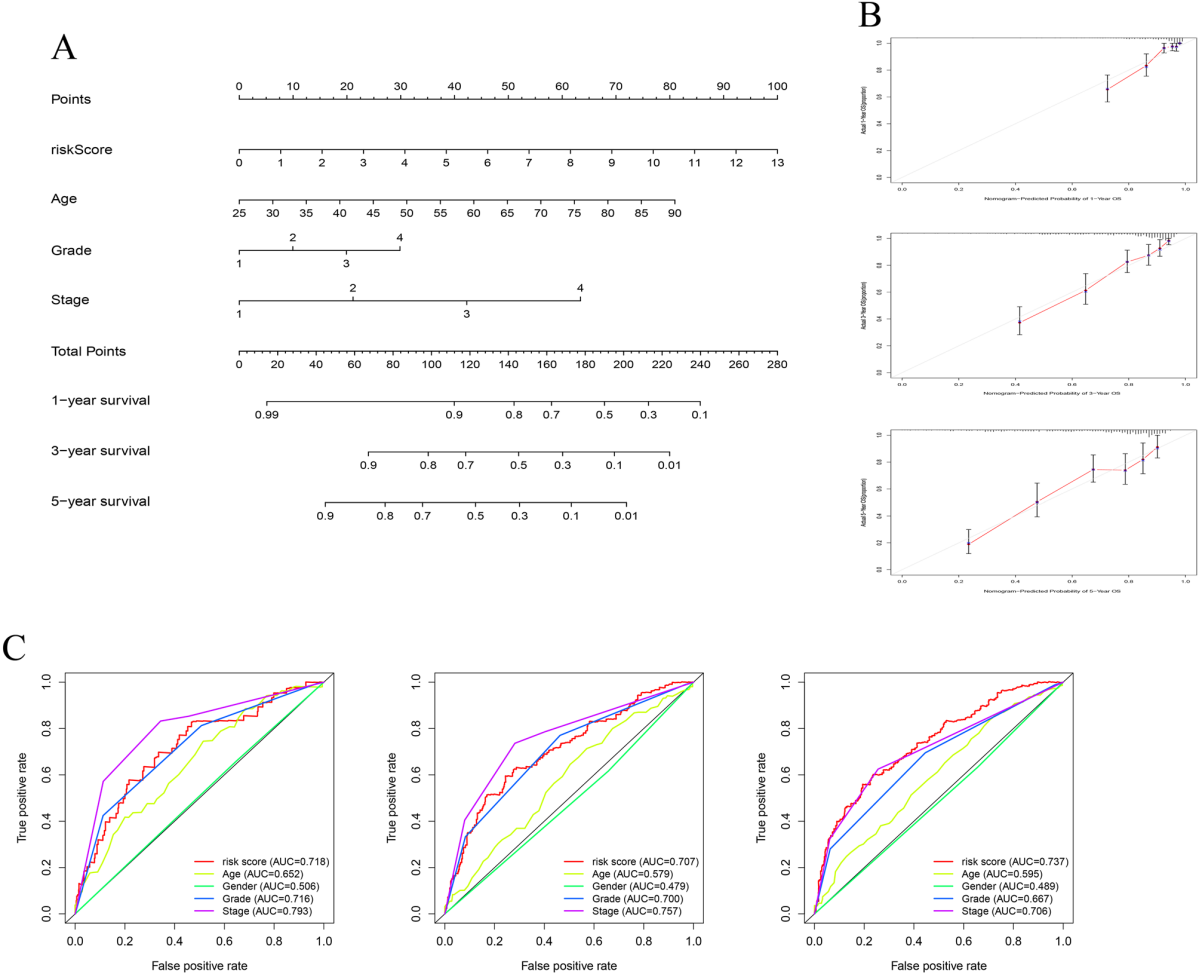
Construction of a nomogram for predicting the survival of KIRC patients. (**A**) A prognostic nomogram that included clinical pathological features (age, stage, risk score), predicting the 1-, 3- and 5-year survival rates of KIRC patients. (**B**) The calibration plots for 1-, 3- and 5-year OS show the consistency between the survival probability predicted by the nomogram and the actual outcome. (**C**) The receiver operating characteristic of OS in 1-, 3- and 5-year showed the prognostic accuracy of anoikis-related gene risk score and other clinical characteristics.

In order to accurately predict the OS of KIRC patients, we constructed nomogram of 1-, 3- and 5-year survival probabilities based on risk scores and clinical pathological characteristics (Fig. 4A). The results of the calibration curve show that the actual OS was basically consistent with the OS predicted through the nomogram (Fig. 4B). According to the results of receiver operating characteristic (ROC), the AUC value of anoikis-related signature was superior to other clinical factors, such as age, sex and histological grade, but inferior to the clinical stage in 1-, 3-year OS (Fig. 4C).

### Analysis of the immune microenvironment (TME) and immune-related pathways

**Figure 5:**
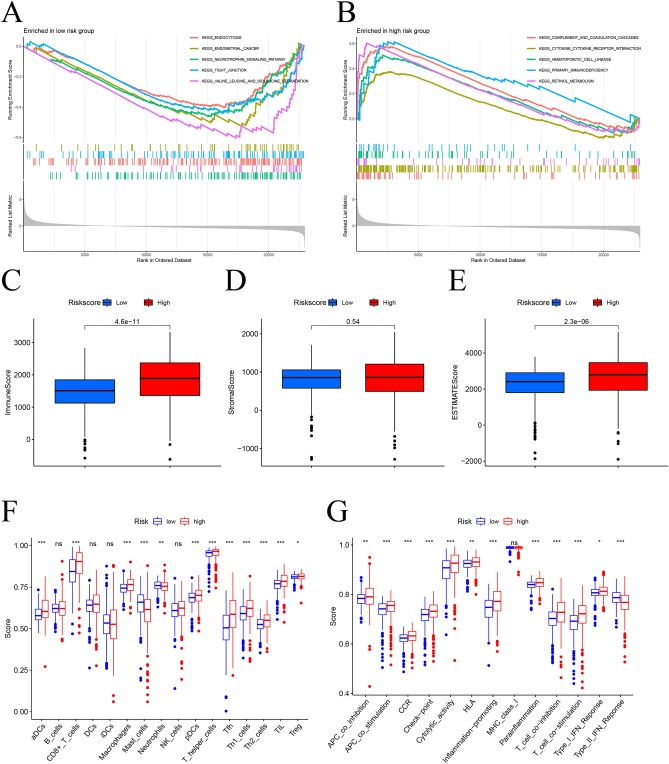
Gene set enrichment analysis and estimation of immune cell infiltration in different risk groups. GSEA analysis was used to predict potential functions and pathways in high-risk (**A**) and low-risk (**B**) groups. (**C**–**E**) The stromal score, immune score, and ESTIMATE score were detected in different risk groups. (**F**,**G**) Differences in immune cells and immune-related functions between the two risk groups. *ns* not significant, *p < 0.05, **p < 0.01, ***p < 0.001.

To elucidate the potential tumor related pathways between the LR and HR groups, GSEA analysis was conducted. The results showed that the HR group mainly enriched in complement and coagulation cascades, cytokine and cytokine receptor interaction, while the LR group was mainly related to pathways with endocytosis, endometrial cancer (Fig. 5A,B). Afterwards, we used ESTIMATE to calculate the proportion of stromal cells and immune cells in different risk groups to estimate the purity of tumors. The HR group had higher immune scores, while the tumor purity was relatively low (Fig. 5C–E). The results of ssGSEA confirmed that the HR group had higher levels of immune cell infiltration and more active immune related functions (Fig. 5F,G). These findings indicate that patients in the HR group, although with poor prognosis, have strong immunity and may be more sensitive to immunotherapy.

**Figure 6:**
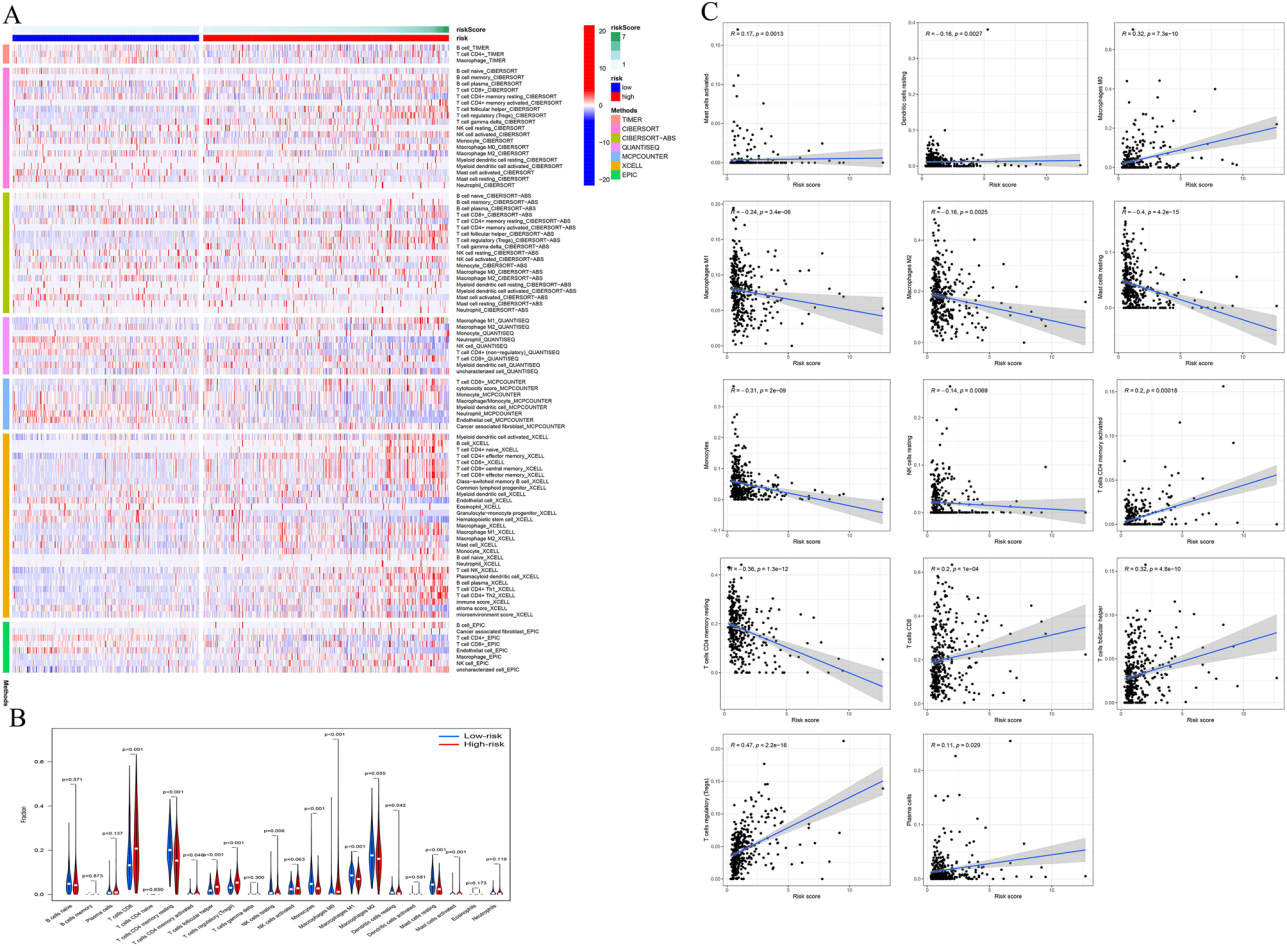
Correlation between tumor infiltrating immune cells (TIC) and risk score. (**A**) The infiltration of 21 immune cells in high-risk and low-risk populations was evaluated using different database such as TIMER, CIBERSORT, CIBERSORT-ABS, QUANTISSEQ, MCPCOUNTER, XCELL, and EPIC databases. (**B**) Comparison of TIICs between high-risk and low-risk groups. (**C**) The correlation between risk score and the degree of immune cell infiltration.

We further explored the role of the tumor microenvironment in KIRC patients with different risk scores. Algorithms such as TIMER, CIBERSORT and EPIC were used to explore the expression levels of tumor immune infiltrating cells (TIICs) in different risk groups (Fig. 6A). In addition, CIBERSORT algorithms was used to compare differences in immune infiltration levels of 22 immune cells between different risk groups (Fig. 6B). In the LR group, the proportion of resting CD4 memory T cells, M1 and M2 macrophages, and resting mast cells increased significantly. The infiltration degree of Tregs and M0 macrophages increased significantly in HR group. Finally, we further evaluated the relationship between risk score and TIIC, and the risk score showed a significant positive correlation with Tregs, but a significant negative correlation with mast cells (Fig. 6C). These results indicate that anoikis-related signature can effectively distinguish different features of TIIC in KIRC patients.

### Somatic variation analysis

**Figure 7:**
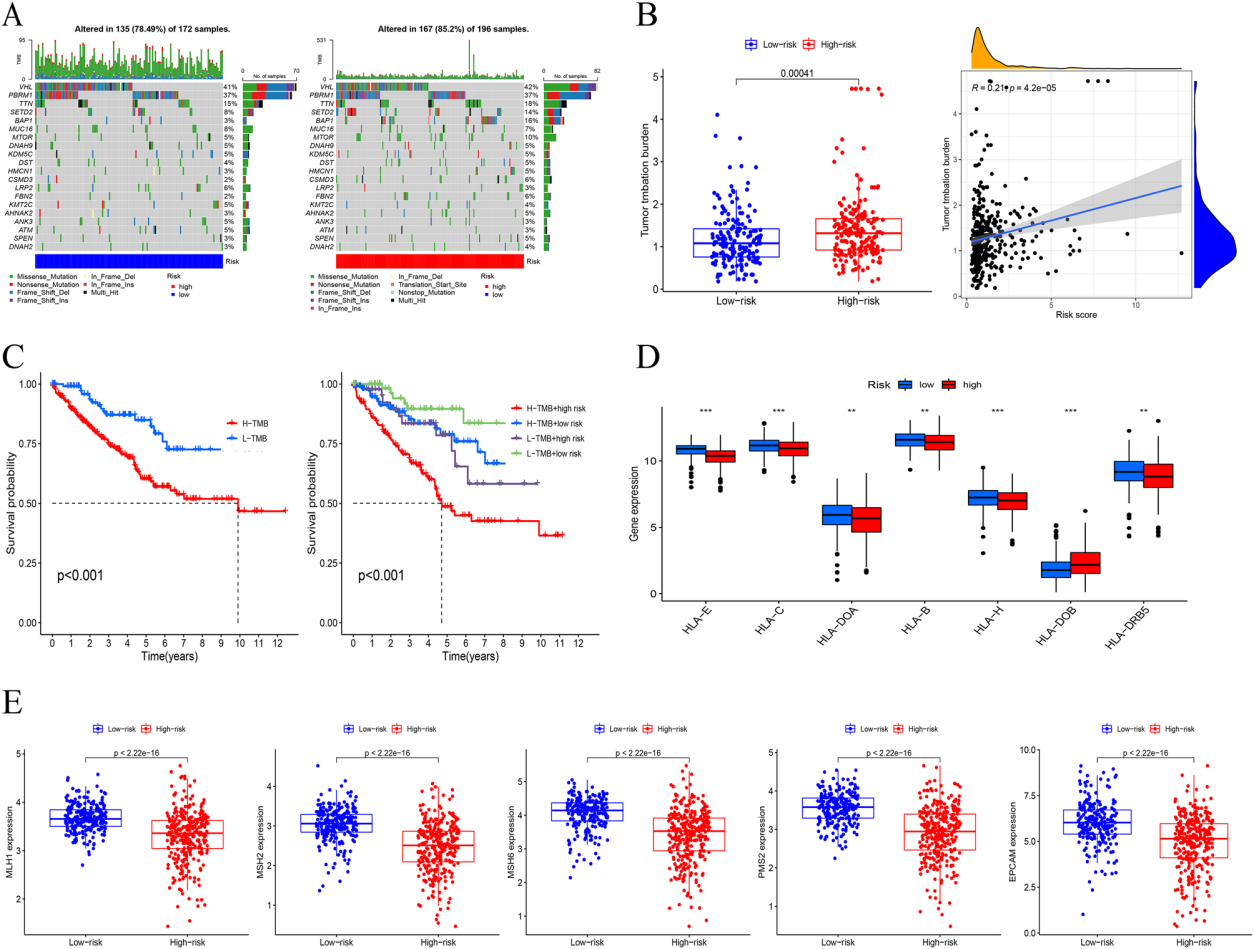
Tumor mutation burden and mutation analysis. (**A**) The waterfall plot revealed the mutation information of genes with high mutation frequency in the high-risk and low-risk groups. (**B**) The difference in TMB between high-risk and low-risk groups and the correlation between TMB and risk score. (**C**) The survival curves of patients in different risk groups and TMB groups. (**D**) The expression of HLA-related genes in two risk groups. (**E**) The expression of MLH1, MSH2, MSH6, PMS2, and EPCAM in two risk groups.

Other study suggested that patients with higher TMB may benefit from immunotherapy due to a higher number of antigens. We generated two waterfall plots to explore the detailed gene mutations between the LR and HR groups (Fig. 7A). Afterwards, we investigated the correlation between risk score and TMB level, as shown in Fig. 7B. The TMB level in the LR group was significantly lower, and there was a positive correlation between TMB and risk score. According to the median TMB values, patients were divided into two groups for survival analysis. The combination of risk score and TMB was used to divide patients into four subgroups for survival evaluation. The results showed that the low TMB and LR groups had the best prognosis, which helps to screen the best prognostic subgroups for clinical use (Fig. 7C). HLA is widely used in the research of immune related diseases, organ and bone marrow transplantation, vaccine and drug targeted population screening, tumor immune research. Next, we evaluated the expression differences of HLA-related genes in different risk groups (Fig. 7D). In addition, expression level mismatch repair (MMR)-related genes were detected, and the results showed that MLH1 | EPCAM, MSH2, PMS2, and MSH6 were downregulated in the low-risk group (Fig. 7E).

**Figure 8:**
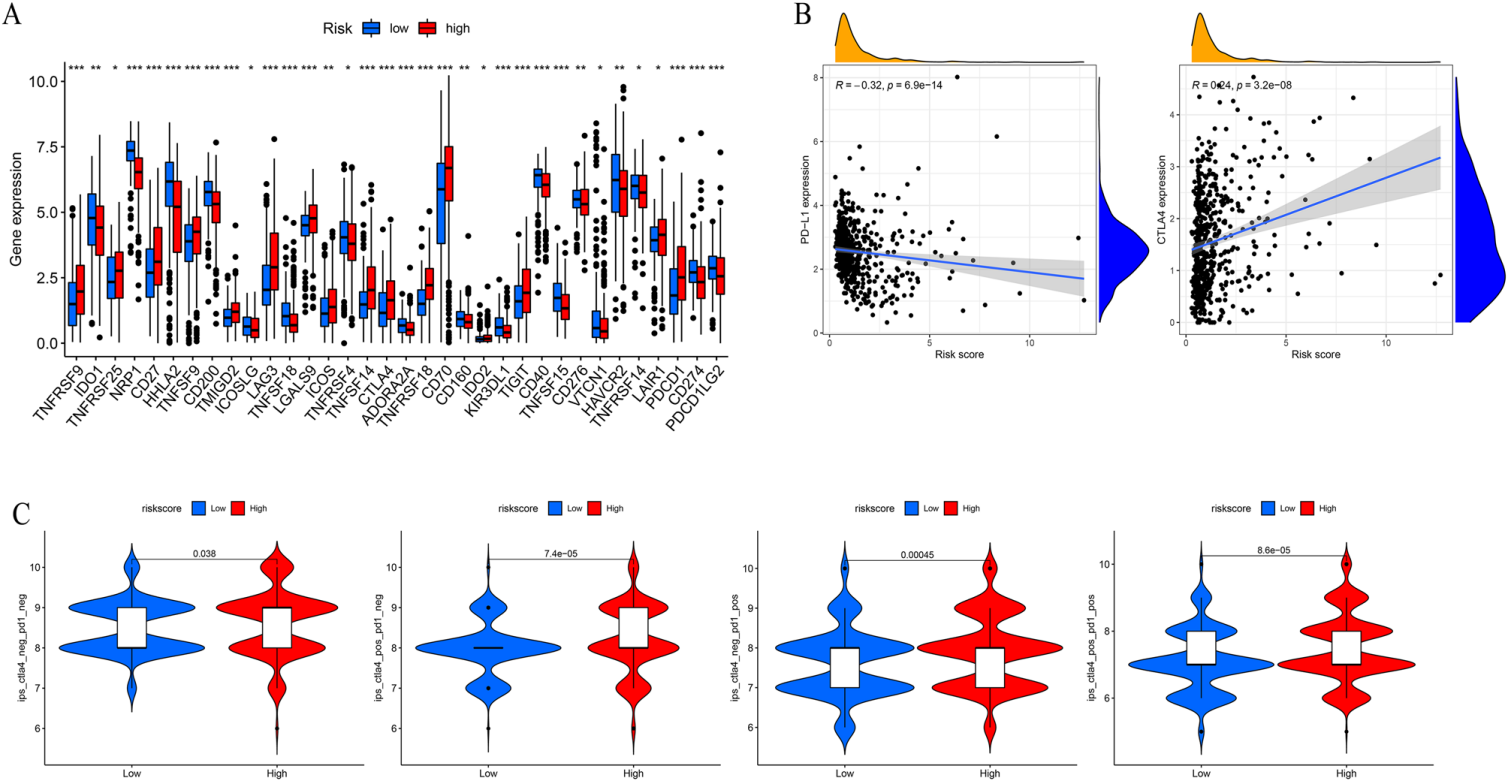
Results of immune checkpoint and immunophenotype (IPS) analysis. (**A**,**B**) The expression of immune checkpoint-related genes and the correlation between risk scores and CTLA4 expression and PD-L1 expression. (**C**) Differences in IPS among patients with different risk scores. ns, not significant, *p < 0.05, **p < 0.01, ***p < 0.001.

We found significant differences in the vast majority of immune checkpoint genes between the two risk groups. Most of them were typically significantly higher in the HR group (Fig. 8A). In addition, the risk score was positively correlated with CTLA4 expression and negatively correlated with PD-L1 (Fig. 8B). The immunophenotype (IPS) quantitative scoring scheme could be used to determine the determining factors of tumor immunogenicity and serve as an effective predictor for detecting anti PD-1 and anti CTLA4 antibody responses. To evaluate the likelihood of receiving immune checkpoint inhibitor (ICB) treatment, we calculated the IPS score. As shown in Fig. 8C, the HR group had higher IPS scores. The results demonstrate the effectiveness of anoikis-related prognostic signatures in predicting immunotherapy.

### Chemotherapy sensitivity analysis

**Figure 9:**
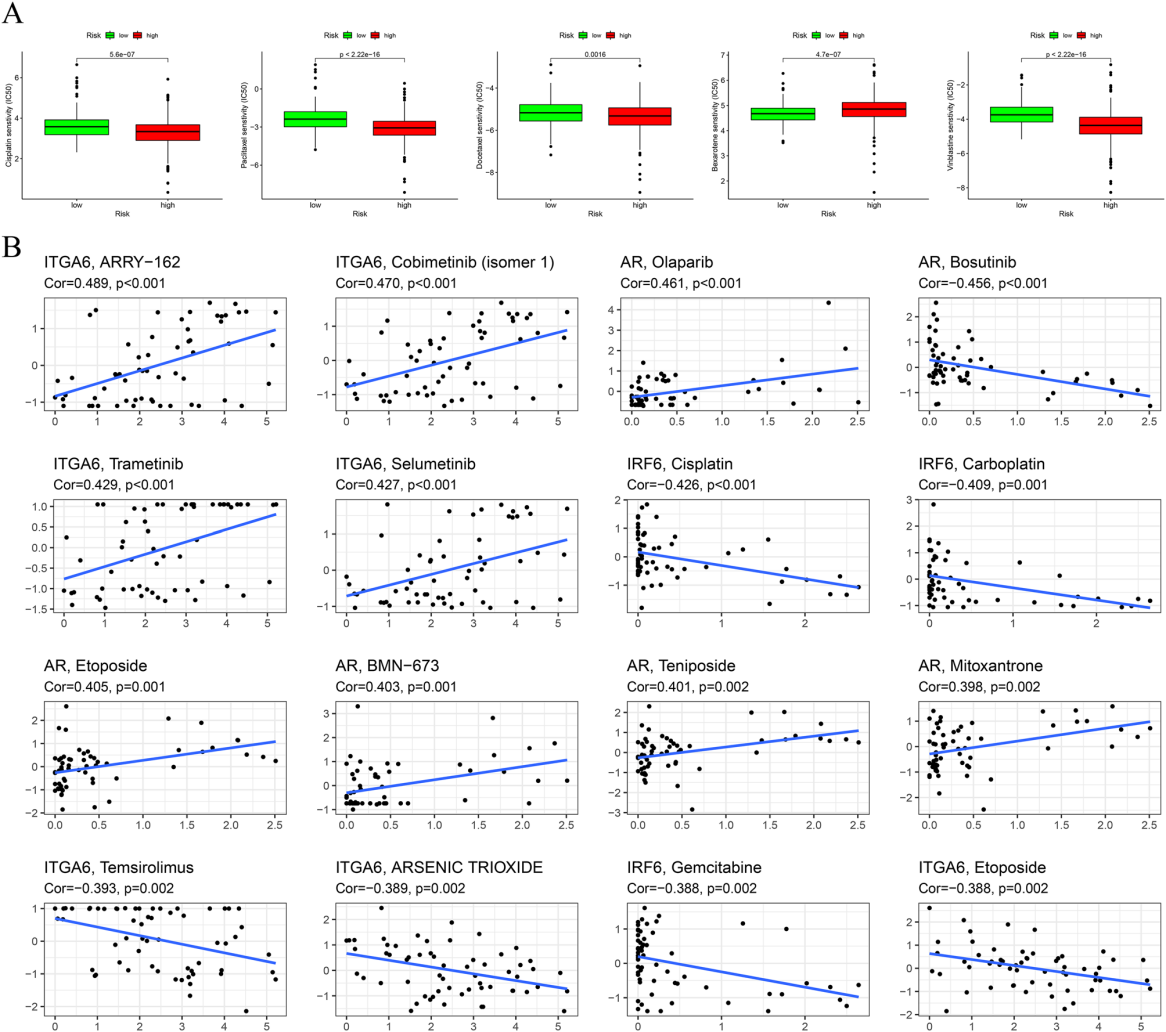
Chemotherapy drug analysis. (**A**) Estimates of IC50 values for cisplatin, paclitaxel, docetaxel, bexarotene, and vincristine in different risk groups. (**B**) Scatter plots of the association between four ARGs expression and drug sensitivity.

To further explore differences in chemotherapy drug resistance, we compared IC50 levels of chemotherapy drugs including cisplatin, paclitaxel, docetaxel, bexarotene, and vincristine. The HR group was more sensitive to cisplatin, paclitaxel, docetaxel, and vinblastine (Fig. 9A). In addition, a strong association was found between the expression of four ARGs and the sensitivity of some chemotherapy agents (Fig. 9B). For example, the expression level of IRF6 was positively correlated with cisplatin sensitivity.

## Discussion

Renal clear cell carcinoma (ccRCC) is a widespread form of kidney cancer, accounting for a significant proportion^35^. This type of cancer is typified by the presence of clear cells in the tumor tissue, and is notorious for its aggressive behavior and resistance to conventional therapies. Anoikis, a type of apoptosis induced by the detachment of cells from their extracellular matrix (ECM), is critical in maintaining tissue homeostasis and eliminating abnormal or damaged cells^36^. Typically, separation from the ECM triggers anoikis, leading to apoptosis and cell death. However, recent studies have demonstrated that ccRCC cells exhibit a high level of resistance to anoikis, enabling them to evade the normal cell death process and sustain tumor cell survival. This resistance to anoikis is thought to play a vital role in the development and progression of ccRCC. In particular, ccRCC cells have been found to express high levels of anti-apoptotic proteins, such as Bcl-2 and survivin, which hinder anoikis and sustain tumor cell survival^37,38^. In sum, the resistance to anoikis is a hallmark of ccRCC and holds a crucial role in its development and progression. Decoding the molecular mechanisms underlying anoikis regulation in ccRCC cells holds enormous potential for the development of new and more effective treatments for this cancer.

In this study, we integrated the anoikis gene expression profile from the TCGA-KIRC dataset and selected four genes to construct a novel anoikis-related prognostic model using Lasso regression analysis and COX hazard regression analysis. The anoikis-related prognostic model was demonstrated to be an independent prognostic factor for ccRCC and was divided into two different prognostic subgroups based on the median risk score. Subsequently, ROC curves, precision-recall plots, and calibration curves were constructed and a comprehensive analysis indicated that the predictive performance of the prognostic model was more pronounced compared to other conventional clinical indicators such as age, grade, and stage. Furthermore, there was satisfactory consistency between the predicted values and the observed values. This can provide theoretical basis for clinical decision-making by medical practitioners.

ITGA6, or integrin alpha 6, is a cell surface receptor that plays a crucial role in cell adhesion, migration and survival^39^. Recent studies have shown that ITGA6 is dysregulated in various types of cancer, including ccRCC. The abnormal expression of ITGA6 in ccRCC cells has been implicated in promoting tumor cell survival, angiogenesis and resistance to apoptosis. The role of Androgen Receptor (AR) in the development and progression of ccRCC is complex and still not fully understood. In ccRCC, AR expression has been shown to be associated with increased cell proliferation and decreased apoptosis, which can lead to the development of the cancer^40^. These findings highlight the potential of AR as a therapeutic target for ccRCC, but further studies are necessary to fully understand the mechanisms underlying the interaction between AR, ccRCC, and anoikis. PLK1, also known as Polo-like kinase 1, is a serine/threonine kinase that is thought to be involved in the regulation of various cellular processes such as cell division, DNA repair, and cell survival^41^. In ccRCC, overexpression of PLK1 has been observed and is considered a potential therapeutic target for treating this invasive cancer^42^. In ccRCC cells, overexpression of PLK1 has been shown to confer resistance to anoikis, making it a potential factor in the development and progression of ccRCC. IRF6 is a transcription factor that plays a crucial role in the regulation of cellular processes such as development and immune response^43^. The alteration of IRF6 expression has been shown to contribute to the progression and metastasis of ccRCC by impacting the regulation of genes involved in cell survival and apoptosis. Additionally, IRF6 has been implicated in the resistance of ccRCC cells to anoikis, a form of apoptosis that occurs when cells are detached from their extracellular matrix^44^. These findings suggest that IRF6 may represent a potential therapeutic target for the treatment of ccRCC.

RCC stands out as one of the most immune-infiltrated tumors in pan-cancer comparisons^45,46^. The tumor microenvironment features with extensive angiogenesis and inflammatory features show significant differences in response to immune checkpoint blockade and anti-angiogenic drugs^47,48^. Therefore, the integration of tumor microenvironment and immune biomarkers can generate predictive and prognostic features to guide the management of existing protocols and future drug development^49–51^. In addition, Analyzing the tumor microenvironment can infer the effectiveness of immunotherapy. The crucial importance of understanding the role of anoikis in the microenvironment of ccRCC and finding ways to overcome its resistance has been highlighted for the advancement of more effective treatments. Immunotherapy, which works by activating the immune system to attack and eliminate cancer cells, has shown promise in treating ccRCC^52^. However, ccRCC cells often exhibit resistance to anoikis, limiting the effectiveness of immunotherapy. To address this issue, researchers are exploring the molecular mechanisms behind anoikis resistance and developing strategies to target these mechanisms, such as targeting specific proteins or pathways that suppress anoikis^53^. By inducing anoikis in ccRCC cells or increasing their sensitivity to anoikis, it is believed that the number of cancer cells can be reduced, leading to improved efficacy of ccRCC treatment and better patient outcomes. The restoration of anoikis in cancer cells and improvement of immunotherapy through these means holds promise as a promising approach for the treatment of ccRCC. In our study, we found a higher degree of immune infiltration in the high-risk group, which also implies that patients in the high-risk group are more suitable for the application of immunotherapy.

TMB has become a promising biomarker in the field of cancer research, particularly for ccRCC^54^. TMB refers to the number of mutations present in the cancer cells of a patient and is seen as an indicator of the immune system's ability to recognize and attack the cancer cells^55^. High TMB is associated with increased sensitivity to immunotherapy, making it a useful predictor of patient response to immunotherapy^56^. Studies have shown that patients with ccRCC who have high TMB tend to have a better response to immunotherapy compared to those with low TMB^57^. This is due to the presence of more mutations in the cancer cells, which in turn leads to a stronger immune response and a higher likelihood of success with immunotherapy. This finding has led to the development of TMB-based clinical trials and the incorporation of TMB into treatment decision-making for ccRCC patients. Additionally, research has also shown that TMB may be an independent predictor of overall survival in ccRCC patients^58^. Patients with high TMB have been found to have a better prognosis and improved survival outcomes compared to those with low TMB^59,60^. Our results also showed that patients in the high-risk group had a higher TMB than those in the low-risk group, implying that patients in the high-risk group are more likely to benefit from immunotherapy. This highlights the importance of TMB in guiding treatment decisions and improving patient outcomes for ccRCC patients. TMB has emerged as a promising biomarker in the field of ccRCC research. Its ability to predict patient response to immunotherapy and overall survival has led to its increasing use in clinical decision-making and its incorporation into clinical trials for ccRCC. Further research is needed to fully understand the implications of TMB in ccRCC and its potential as a therapeutic target.

Our study has clinical significance for prognostic assessment and treatment selection for ccRCC patients, yet it has some limitations. It is a retrospective study that needs to be validated in prospective studies. The signature's potential to predict immunotherapy response was assessed indirectly due to lack of mRNA expression profile data from ccRCC patients receiving immunotherapy, which may lead to discrepancies. Thus, future validation should be done with data from ccRCC patients receiving immunotherapy.

### Supplementary Information

Supplementary Figure S1.

Supplementary Figure S2.

Supplementary Figure S3.

Supplementary Information.

## Data Availability

The dataset generated and analyzed during the current study are available in The Cancer Genome Atlas (TCGA) with TCGA-KIRC accession number and with the link of https://portal.gdc.cancer.gov/.
