# Supplementary figures and images for: Identification of anoikis-related subtypes and immune landscape in kidney renal clear cell carcinoma
Source: Sci Rep. 2023 Oct 23;13:18069. doi: 10.1038/s41598-023-45069-4 (PMC10593771; doi:10.1038/s41598-023-45069-4)

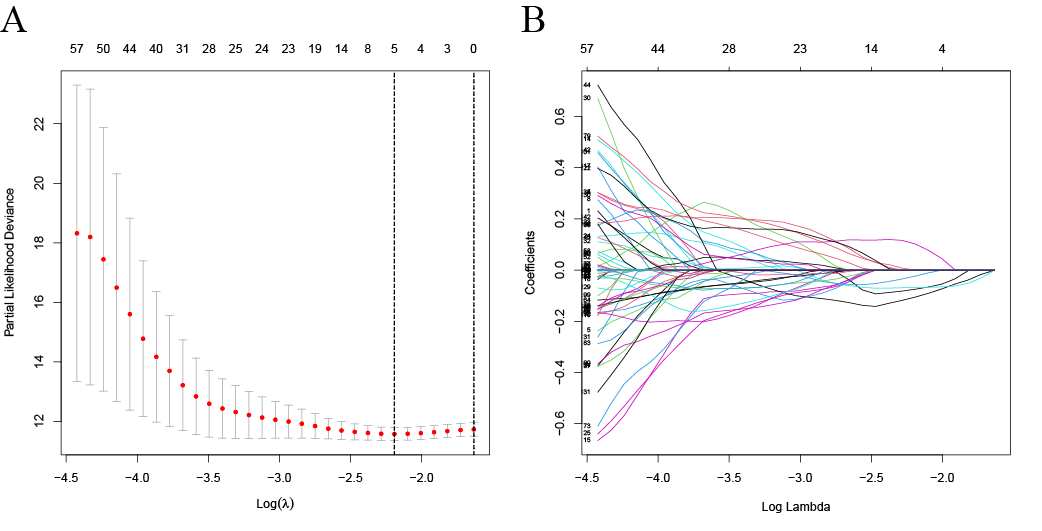

Supplement: Supplementary file 1 — Supplementary Figure S1. [file 41598_2023_45069_MOESM1_ESM.tif]

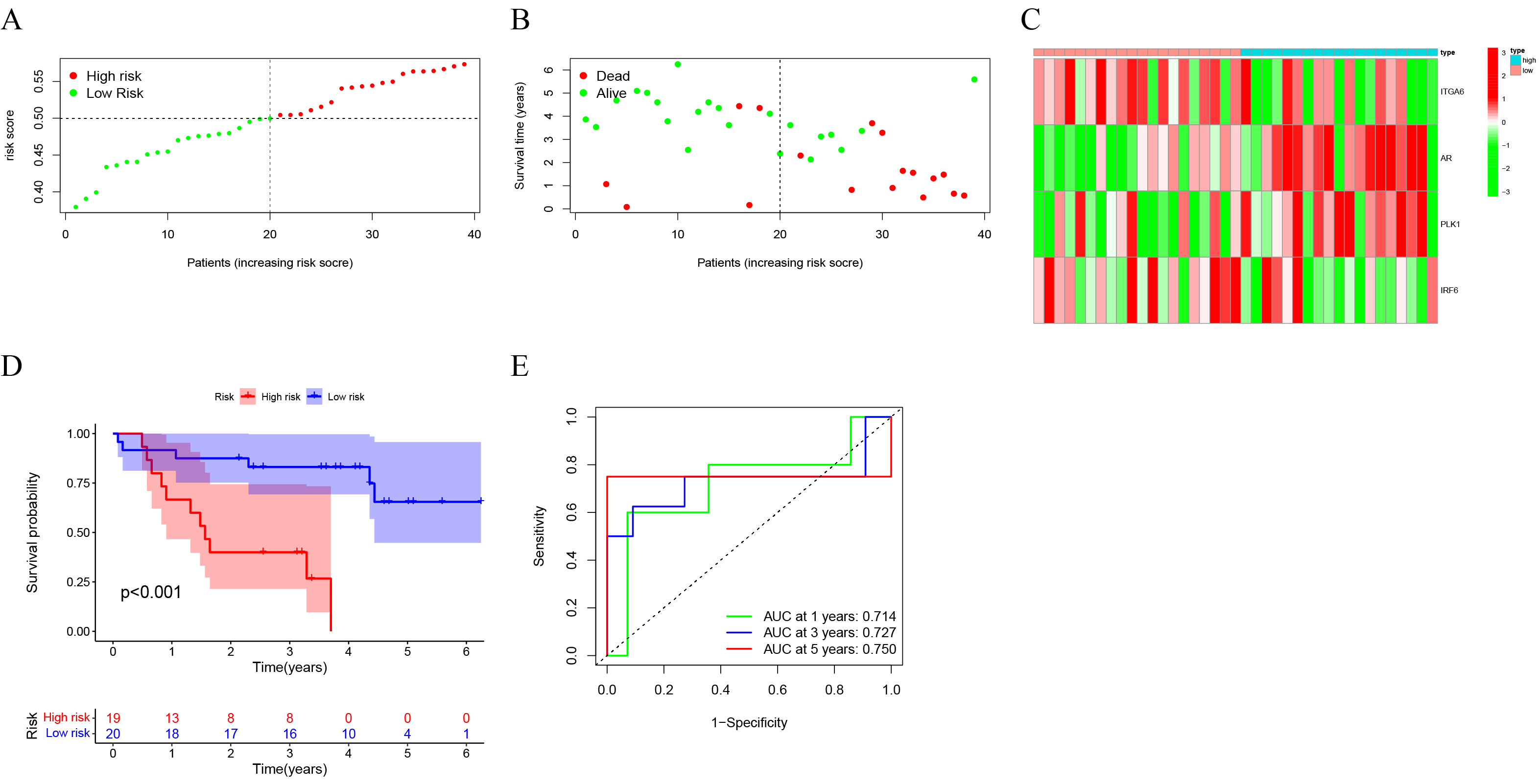

Supplement: Supplementary file 2 — Supplementary Figure S2. [file 41598_2023_45069_MOESM2_ESM.tif]

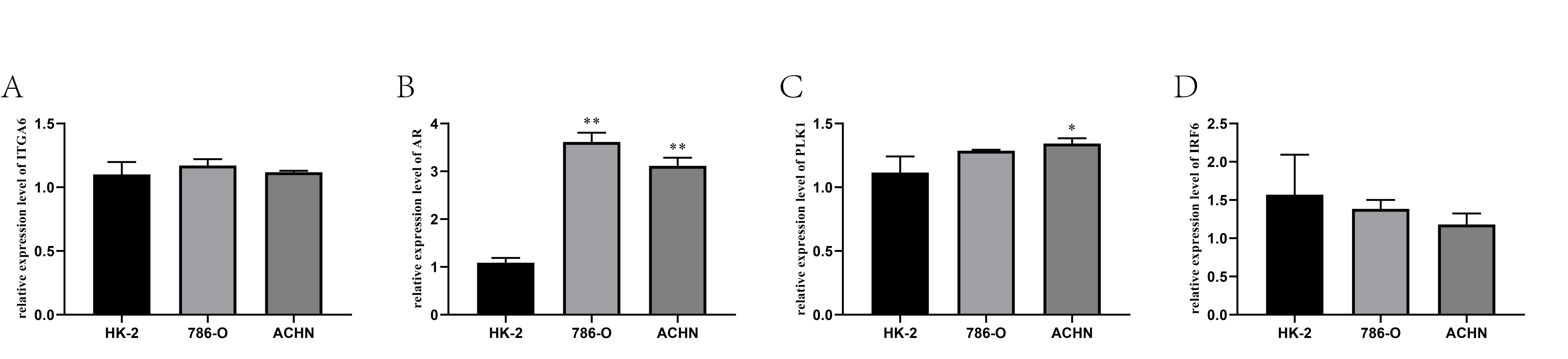

Supplement: Supplementary file 3 — Supplementary Figure S3. [file 41598_2023_45069_MOESM3_ESM.tif]
