# Supplementary material for: Identification of anoikis-related subtypes and immune landscape in kidney renal clear cell carcinoma
Source: Sci Rep. 2023 Oct 23;13:18069. doi: 10.1038/s41598-023-45069-4 (PMC10593771; doi:10.1038/s41598-023-45069-4)
Supplement: Supplementary file 4 — Supplementary Information. [file 41598_2023_45069_MOESM4_ESM.docx]

Supplementary Figure 1. Multivariate analysis and lasso regression analysis. (A, B) 5 ARGs were chosen for multivariate analysis based on the outcomes of lasso regression analysis.

Supplementary Figure 2. Validation of anoikis-related signatures in GEO cohort. (A, B) The risk score and survival state distribution. (C) Heat map of four candidate gens expression. (D, E). K-M curves and time-dependent ROC curve for OS in GEO cohort.

Supplementary Figure 3. The expression of ARGs in HK2 and two ccRCC cell lines by qRT-PCR analysis. (A) ITGA6, (B) AR, (C) PLK1, (D) IRF6. * p < 0.05, ** p < 0.01.

Supplementary Table 1 Primer sequence of genes in qRT-PCR.

|  | Forward sequence | Reverse sequence |
| --- | --- | --- |
| ITGA6 | ATGCACGCGGATCGAGTTT | TTCCTGCTTCGTATTAACATGCT |
| AR | CCAGGGACCATGTTTTGCC | CGAAGACGACAAGATGGACAA |
| PLK1 | AAAGAGATCCCGGAGGTCCTA | GGCTGCGGTGAATGGATATTTC |
| IRF6 | CCCCAGGCACCTATACAGC | TCCTTCCCACGGTACTGAAAC |
| GAPDH | GGAGCGAGATCCCTCCAAAAT | GGCTGTTGTCATACTTCTCATGG |

Supplementary Table 2 Distribution of patients into training cohort, testing cohort and total cohort.

|  | Type | Total | Test | Train | Pvalue |
| --- | --- | --- | --- | --- | --- |
| Age | <=65 | 349(65.48%) | 175(65.79%) | 174(65.17%) | 0.9524 |
|  | >65 | 184(34.52%) | 91(34.21%) | 93(34.83%) |  |
| Gender | FEMALE | 188(35.27%) | 97(36.47%) | 91(34.08%) | 0.6275 |
|  | MALE | 345(64.73%) | 169(63.53%) | 176(65.92%) |  |
|  | G1 | 14(2.63%) | 6(2.26%) | 8(3%) | 0.36 |
|  | G2 | 229(42.96%) | 100(37.59%) | 129(48.31%) |  |
|  | G3 | 206(38.65%) | 113(42.48%) | 93(34.83%) |  |
| Grade | G4 | 84(16.76%) | 47(17.68%) | 37(13.86%) |  |
|  | Stage I | 267(50.09%) | 129(48.5%) | 138(51.69%) | 0.6178 |
|  | Stage II | 57(10.69%) | 28(10.53%) | 29(10.86%) |  |
|  | Stage III | 123(23.08%) | 61(22.93%) | 62(23.22%) |  |
| Stage | Stage IV | 86(16.13%) | 48(18.05%) | 38(14.23%) |  |
|  | T1 | 273(51.22%) | 132(49.62%) | 141(52.81%) | 0.4567 |
|  | T2 | 69(12.95%) | 35(13.16%) | 34(12.73%) |  |
|  | T3 | 180(33.77%) | 91(34.21%) | 89(33.33%) |  |
| T | T4 | 11(2.06%) | 8(3.01%) | 3(1.12%) |  |
|  | M0 | 422(79.17%) | 206(77.44%) | 216(80.9%) | 0.3867 |
| M | M1-X | 111(20.83%) | 60(22.56%) | 51(19.09%) |  |
|  | N0 | 240(45.03%) | 121(45.49%) | 119(44.57%) | 0.9766 |
| N | N1-X | 293(54.97%) | 145(54.51%) | 148(55.43%) |  |
